# Team-based learning on a third-year pediatric clerkship improves NBME subject exam blood disorder scores

**DOI:** 10.3402/meo.v20.29021

**Published:** 2015-10-13

**Authors:** Kris Saudek, Robert Treat

**Affiliations:** 1 Department of Pediatrics, Medical College of Wisconsin, Milwaukee, WI, USA; 2 Pediatric Residency Program, Medical College of Wisconsin, Milwaukee, WI, USA; 3 Department of Emergency Medicine, Medical College of Wisconsin, Milwaukee, WI, USA; 4 Office of Academic Affairs, Medical College of Wisconsin, Milwaukee, WI, USA

**Keywords:** curriculum, medical students, performance on examinations, teamwork, clinical application

## Abstract

**Purpose:**

At our institution, speculation amongst medical students and faculty exists as to whether team-based learning (TBL) can improve scores on high-stakes examinations over traditional didactic lectures. Faculty with experience using TBL developed and piloted a required TBL blood disorders (BD) module for third-year medical students on their pediatric clerkship. The purpose of this study is to analyze the BD scores from the NBME subject exams before and after the introduction of the module.

**Methods:**

We analyzed institutional and national item difficulties for BD items from the NBME pediatrics content area item analysis reports from 2011 to 2014 before (pre) and after (post) the pilot (October 2012). Total scores of 590 NBME subject examination students from examinee performance profiles were analyzed pre/post. *t*-Tests and Cohen's *d* effect sizes were used to analyze item difficulties for institutional versus national scores and pre/post comparisons of item difficulties and total scores.

**Results:**

BD scores for our institution were 0.65 (±0.19) compared to 0.62 (±0.15) nationally (*P*=0.346; Cohen's *d*=0.15). The average of post-consecutive BD scores for our students was 0.70 (±0.21) compared to examinees nationally [0.64 (±0.15)] with a significant mean difference (*P*=0.031; Cohen's *d*=0.43). The difference in our institutions pre [0.65 (±0.19)] and post [0.70 (±0.21)] BD scores trended higher (*P*=0.391; Cohen's *d*=0.27). Institutional BD scores were higher than national BD scores for both pre and post, with an effect size that tripled from pre to post scores. Institutional BD scores increased after the use of the TBL module, while overall exam scores remained steadily above national norms.

**Conclusions:**

Institutional BD scores were higher than national BD scores for both pre and post, with an effect size that tripled from pre to post scores. Institutional BD scores increased after the use of the TBL module, while overall exam scores remained steadily above national norms.

Team-based learning (TBL) is an educational approach that harnesses the benefits of small group work and promotes active learning (1). TBL relies on student preparation of instructor-assigned materials prior to the TBL session in class. During class time, work is completed in small groups of students with assessment of acquisition knowledge at the beginning of the formal TBL session. In active learning sessions, the majority of class time is spent on instructor-crafted application exercises that require learners to apply the knowledge acquired in pre-class preparation to real-world, high-stakes problems and clinical scenarios. Active learning has established itself as a more powerful technique than passive learning, and the Liaison Committee for Medical Education (LCME) has required that medical education ‘includes self-directed learning experiences and time for independent study to allow medical students to develop the skills of lifelong learning’ (2, 3). After successful piloting of using self-directed and active learning in undergraduate medical education curricula, many medical schools are now using TBL to address this paradigm shift in education in the preclinical years (4, 5). While it is encouraging to see that TBL is increasingly being incorporated in the preclinical curriculum, there is very little evidence in the literature that TBL is being utilized in the clinical years.

Students have indicated that they prefer self-directed, hands-on learning over traditional passive didactic lectures making TBL an appropriate educational process for engaging and educating students today (6). Many studies have reported that students uniformly enjoyed their TBL experience (7–9). However, student skepticism regarding this method of teaching is documented in the literature as well, even in the studies that showed positive outcomes of TBL on student performance (10, 11). While student evaluation of curriculum and satisfaction with their education is important because it fosters their enthusiasm in the process, reaction data are still an indirect measure of actual learning outcomes. There are conflicting reports on whether TBL improves outcomes over traditional didactic lectures (8, 12, 13). One study reported that students performed well on course assignments related to a TBL session, but did not demonstrate that it was more effective than having a traditional lecture in achieving these goals (14). Another study demonstrated improved knowledge retention using TBL over passive learning up to 48 h later, but longer term acquisition of knowledge was not addressed (15).

Educators need to be diligent about publishing their experiences and results using TBL and how it impacts performance on high-stakes examinations (16). Additionally, its use in the clinical years of medical school should not be overlooked. Clinical reasoning and collaboration with colleagues has been enhanced using TBL in the clinical years (17, 18). These skills are critical to develop over the course of a career medicine and should span all years of medical education. This is particularly important regarding data in the medical literature that unprofessional behavior in medical school is associated with unprofessional behavior later in one's career (19). TBL in the context of professional development seems particularly appealing given a study that reported the most common report of unprofessional behavior in medical students was an inability to get along with members of the healthcare team (20).

The aim of this study was twofold. First, we wanted to assess changes in the blood disorder (BD) content area scores from the NBME pediatrics subject exam as a result of the implementation of a TBL session developed for students on the third-year pediatric clerkship. We hypothesized that this TBL intervention would increase NBME pediatrics subject exam BD scores for third-year medical students. Second, we wanted to pilot TBL in the clinical years at our institution, highlighting its use not only in the context of learning but also in personal and professional development.

## Methods

In October 2012, we piloted a TBL BDs module on the third-year pediatric clerkship. The module was created by faculty members with experience using TBL and was published in MedEdPortal (21). The faculty considered topics that could be challenging for students to apply clinically and chose BDs.

All third-year medical students rotate through an 8-week pediatric clerkship of approximately 32–36 students per cohort. The session was given every month to 16–18 students. Pre-class reading highlighting the core content was assigned to the students with the expectation it would be read prior to the session. During the session, teams of 5–6 students were created. We introduced the session as a clinical application exercise and emphasized the collaborative team component mimics hospital team rounding to help students focus on the broader application of this exercise. Formally, the session was listed as BDs on the syllabus, with no indication that it was a TBL session. An individual readiness assurance test (IRAT) consisting of multiple-choice items based on the pre-class reading was administered at the beginning of the session. Next, students collaborated on the group readiness assurance test (GRAT) to arrive at a single best answer. When the team reached a consensus, they used scratch-off immediate feedback (IF-AT) cards (available at www.epsteineducation.com/home/) revealing a star if the correct answer was chosen. A ‘mini’ lecture was given by the faculty based on observations made during the testing portion of the session, and questions from the IRAT and GRAT that posed difficulty were discussed more thoroughly. A series of application exercises made up the bulk of the session. These real-world, high-stakes clinical scenarios had several reasonable answers but one that was better than the rest. When teams arrived at a consensus, the facilitator asked each group to discuss their response. Often, teams had arrived at different answers so the facilitator guided a discussion among the groups.

Data were analyzed from the NBME pediatrics subject examination content area item analysis (CAIA) reports and examinee performance profiles (EPP). The CAIA reports the content area classification for each item on the subject examination and reports institutional and national item difficulty scores and their difference to each medical school. Items classified as ‘diseases of the blood’ were identified and their corresponding item difficulty values were analyzed: 1) institutionally versus nationally, and 2) before (pre) pilot program (February 2011 to August 2012, *N*=334 students versus after (post) pilot program (December 2012 to February 2014, *N*=256 students). The EPP reports the total scaled scores for each student whereby the entire set of national scores are standardized to a mean=70 and standard deviation=8.

Mean differences were determined with independent *t*-tests. Effect sizes were calculated with Cohen's *d*. Pearson correlations were used to determine the relational strength between paired outcomes. All data were analyzed with IBM^®^ SPSS^®^ 21.0 (Armonk, NY).

## Results

The average of 10 sets of pre-consecutive clerkship BD scores (*N=*334) on the NBME CAIA report for our institution was 0.65 (±0.19) compared to 0.62 (±0.15) for examinees nationally (*P*=0.346, Cohen's *d*=0.15) as reported in Table 1. The average of eight sets of post-consecutive BD scores (*N=*256) for our students was 0.70 (±0.21) compared to examinees nationally [0.64 (±0.15)] in which the mean difference was statistically significant (*P*=0.031, Cohen's *d*=0.43).

**Table 1:** Institution and national mean pre- and post-NBME pediatrics subject examination blood disorder scores

	Mean (±SD)		
		
	Pre	Post	Significance (*P*)
Institution scores	0.65 (±0.19)	0.70 (±0.21)	0.391
National scores	0.62 (±0.15)	0.64 (±0.15)	0.741
Significance (*P*)	0.346	0.031	–

NBME=National Board of Medical Examiners.

**Fig. 1:**
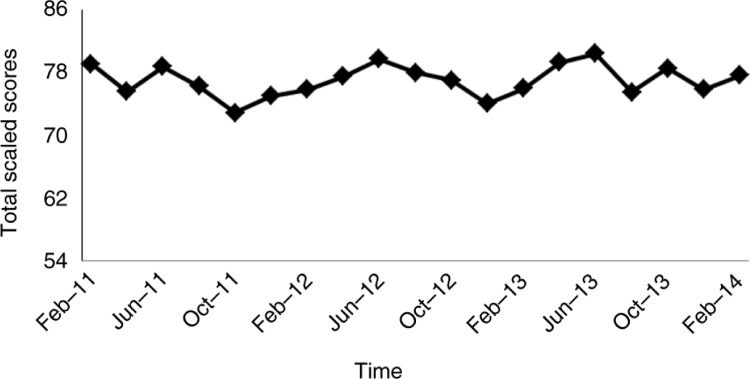
National Board of Medical Examiners pediatrics subject exam total scaled scores across time.

The difference in our institutions pre [0.65 (±0.19)] and post [0.70 (±0.21)] BD scores trended higher albeit was not significant (*P*=0.391, Cohen's *d*=0.27); overall, total scaled scores of 590 subject examination students used as an overall baseline as reported in Fig. 1 were above national levels [70 (±8)] and increased slightly from 76.8 to 77.1 (*P*=0.632, Cohen's *d*=0.04) during the same time periods.

**Fig. 2:**
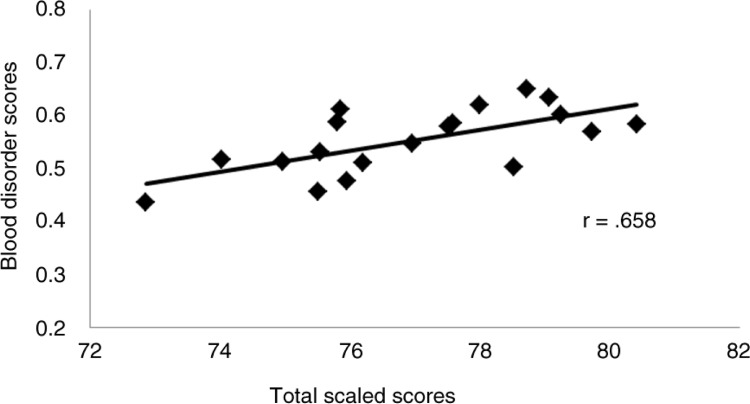
Scatterplot of National Board of Medical Examiners pediatrics subject examination blood disorder scores and total scaled scores.

The correlation of the BD scores and total scaled scores for the entire study was *r=*0.658 (*P*=0.002) with the scatterplot reported in Fig. 2.

## Conclusions

This 3-year study has provided evidence to support the use of TBL in a medical school third-year pediatrics clerkship. BD domain scores from the NBME pediatrics subject exam increased after the implementation of a TBL activity in the clerkship. Generally, NBME total scaled exam scores and BD scores at our institution are higher than the national average. There was an increase in the BD scores from the pre to post half of the study and the overall exam scores followed a similar trajectory.

Faculty and students had expressed some initial reluctance to using alternative learning strategies such as TBL within the clerkship. It has been the experience of educators at our institution that medical students are often skeptical of alternative learning strategies that depart from the traditional didactic lecture platform. This sentiment has been reported by other studies (10, 11, 14). Our goal was to create a specific TBL module relevant to NBME content area ‘diseases of the blood’ and subsequently compare results in this area before and after the pilot session to improve student buy-in of TBL. Review of the medical education literature suggests that this is the first study reporting improved NBME scores on any individual content area using TBL. These are important results as they add to the growing pool of literature that TBL can improve performance on high-stakes examinations.

There are some limitations with this TBL study that should be reported. The use of historical controls makes it difficult to report that TBL was solely responsible for the improvement in scores. Prior to the introduction of the BDs TBL module in the clerkship, there was no formal lecture on this topic in the clerkship and the additional time dedicated to covering this subject alone could account for the improvement in scores.

The clinical experiences and schedule of each student on the clerkship also varied. One of the inpatient teams the students could be assigned to predominantly admit hematology and oncology patients which certainly could have helped them perform better on the NBME examination questions related to BDs. The outpatient clinic experience within the clerkship is also subject to the same variation given the mix of sub-specialty and general pediatric clinics that we assign our students to.

The national mean on standard examinations like the USMLE has been steadily increasing. While the mean standardized score on the NBME pediatrics subject examination is set at 70, over the period of this study we have noticed that the mean percentile ranks of our students have increased. Our students had been exposed to TBL in their pre-clinical years as part of a curriculum overhaul at our institution; however, no specific session on pediatric BDs was part of that curriculum. There was no significant change in our students’ overall NBME pediatrics subject examination score pre- versus post-curriculum but there was a significant change in their BDs scores, which suggests that the prior curriculum did not contribute to the increase in their BD scores. Finally, we did not compare the impact of a didactic lecture in our clerkship to our TBL session, which would be a further area of study.

The future success of TBL at our school is dependent on its acceptance by students and faculty (22). Some authors have reported the importance of demonstrating the effectiveness of TBL prior to investing resources into developing new modules (10). While TBL is easy to facilitate by experienced faculty, the development of the session requires a considerable investment of time. The student evaluations of the session using our institution's standard evaluation form were uniformly positive as were the open-ended comments. It was the highest rated clerkship session for the 2014–2015 academic year at our institution. We did not measure faculty enjoyment of the sessions, but all felt enthused by the experience. In-class interaction between faculty and students appeared more bidirectional, energetic, and rich than with traditional didactic approaches. Since the conclusion of the study, the faculty has acknowledged that the positive exam outcomes will make them consider further review of TBL strategies in other areas of the clerkship.

Students are deeply concerned with their grades and any objective successes of TBL that continue to be published validate its place in education, and there are several concepts from this TBL pilot that may be generalizable to other clerkships. We speculate the initial time we invested with the group while orienting them to the exercise contributed to the positive student evaluations of the session. We did not change the format of TBL as authors have reported the risks of doing so (23). Instead, a small percentage of time was spent describing the advantages of TBL to the students within the context of their personal career and their profession in general. It is important to recognize that medical students may not have the wisdom to recognize being an excellent team player and enjoyable to work with are as important as having great medical knowledge and high test scores. The more educators build the concept of collaboration into the curriculum the more we reinforce it is something we value and expect. It sends a powerful message that students should regard high professional function within a healthcare team as important as studying and accruing medical knowledge. Continuing all these efforts should have a positive impact on student assessment of TBL as a process, and increase student appreciation for TBL, which can help solidify its place in the curriculum.

In conclusion, we found that the addition of a TBL session on BDs resulted in content-specific NBME scores that were significantly higher than the national average. Future efforts will examine how outcomes related to the TBL sessions compare to didactic lectures on the same topic. We also found that faculty and students uniformly enjoyed the sessions. Given this success using TBL at our institution, we are encouraged to utilize it more in the clinical years.
